# Critical Progress of Mn, Cu, Co, and V-MOFs and Their Derivatives as Promising Electrodes for Aqueous Zn-Ion Batteries

**DOI:** 10.3390/nano16010033

**Published:** 2025-12-25

**Authors:** Ramanadha Mangiri, Joonho Bae

**Affiliations:** Department of Semiconductor Physics, Gachon University, Seongnam-si 13120, Republic of Korea

**Keywords:** metal–organic frameworks, zinc-ion batteries, Mn-MOFs, Cu-MOFs, Co-MOFs, V-MOFs

## Abstract

Metal–organic frameworks (MOFs) have emerged as versatile precursors and templates for developing high-performance electrode materials for aqueous zinc-ion batteries (ZIBs), owing to their adjustable porosity, abundant metal-coordination sites, and structural flexibility. Among the diverse array of MOFs investigated, those based on manganese, copper, and cobalt, as well as their derivatives, have shown exceptional potential, exhibiting enhanced redox activity, structural integrity, and advantageous zinc-ion storage kinetics compared with many other MOF systems. This study emphasizes the synthesis methodologies, structural characteristics, and electrochemical benefits of these three significant MOF families. After a succinct overview of MOF chemistry, synthesis methodologies, and fundamental design principles for ZIB electrode materials, the article presents a systematic, comparative evaluation of Mn-MOFs, Cu-MOFs, Co-MOFs and V-MOFs, along with their corresponding metal oxides, sulfides, phosphates, carbon composites, and multidimensional hybrid structures. Recent publications for each MOF type are detailed in separate tables, including synthesis methods, morphological development, electrochemical behavior, and performance metrics. The discourse highlights the distinct properties of each metal center, Mn’s multivalent redox chemistry, Cu’s superior electron transport and coordination adaptability, and Co’s elevated activity and stable structures, which together facilitate improved ion diffusion, substantial reversible capacity, and prolonged cycling durability. Ultimately, existing obstacles and potential research avenues are delineated to advance MOF-based materials for next-generation aqueous ZIB systems.

## 1. Introduction

Rechargeable aqueous zinc-ion batteries (ZIBs) are increasingly recognized as effective large-scale energy storage options, owing to zinc metal’s natural benefits, including a high theoretical capacity (~820 mAh g^−1^), its abundance in the Earth’s crust, and the safety associated with aqueous electrolytes. Nevertheless, several major obstacles hinder their broader adoption: the zinc metal anode faces parasitic side reactions, corrosion, and dendrite formation in aqueous environments, while cathode materials often lack high specific capacity or exhibit poor rate and cycle stability. To address these challenges, electrode materials and interfaces must (i) facilitate fast electron flow and Zn^2+^ mobility during insertion/extraction, (ii) preserve structural integrity over many cycles, and (iii) be scalable and economical to produce. MOFs and related porous structures are promising owing to their high porosity, varied metal–ligand coordination, and ability to form hierarchical, conductive frameworks.

ZIB systems are particularly interested in MOFs because they offer various structural design options, including metal centers, linkers, and pore networks, as well as the ability to transform into derived functional materials, such as metal oxides, sulfides, phosphides, and carbon composites, while maintaining their morphological properties. Consequently, two related yet distinct pathways have emerged: (a) materials made from MOFs that are altered through pyrolysis, sulfidation, or other methods; these produce more porous composites, have improved electrical conductivity, and are more durable over time (for example, MOF → MnOx@C for ZIB cathodes) [1] and (b) pure or conductive MOFs used directly as cathodes or interfacial materials, utilizing open channels for Zn^+^ transport and exposed coordination sites for redox reactions [2]. Researchers have enhanced the capacity, rate capability, and cycle life of aqueous ZIBs using both approaches. This could pave the way for future energy storage solutions. Recent reviews have thoroughly examined ZIB chemistries and electrodes based on MOFS, including methodologies for MOFs in advanced batteries [3], MOF-derived cathodes for hybrid zinc-ion capacitors [4], and studies on carbon-based materials and vanadium oxides for zinc-ion storage [5,6,7]. These works mainly focus on broad cathode categories or specific material families, without providing a framework for comparing different MOF metal centers. This study aims to compare Mn-, Cu-systematically, and Co-MOFs and their derivatives. We unify performance metrics, such as capacity, rate capability, and cycle stability, across these groups, analyze common and unique structural motifs, and develop design principles linking synthesis, nanostructure, and Zn^2+^ storage mechanisms. This comparative method aims to identify design principles that are challenging to discern from isolated or overly broad aqueous ZIB studies.

Focusing specifically on Cu, Mn, and Co as metal centers in MOFs (and their derivatives) is motivated by the distinct redox characteristics, coordination chemistry, and mechanistic opportunities afforded by these transition metals. For example, copper-based MOFs or copper-modified MOF interfaces can provide conductive networks and zincophilic sites to suppress dendrite growth on Zn anodes [8]. Manganese-center MOFs (and derived MnO_x_/carbon composites) are beneficial as cathodes owing to Mn’s multiple oxidation states, high theoretical capacity potential, and the possibility of structural conversion or insertion mechanisms in Zn systems [2]. Cobalt-based MOFs (and bimetallic Co-MOFs) combine robust redox-active centers and structural motifs suitable for Zn^2+^ insertion or surface redox, and thus have been increasingly explored in aqueous Zn systems [9]. Across these metals, design strategies such as heterometal doping, composite formation, carbon-skeleton embedding, 2D sheet morphology, and hierarchical porosity have been introduced to tailor ion/electron transport and structural stability.

In the field of synthesis and structural design, various techniques have been employed, including solvothermal/hydrothermal assembly of MOFs, room-temperature coordination precipitation, electrochemical growth on conductive substrates, and post-treatment methods such as pyrolysis, sulfidation, phosphidation, heterometal doping, or templated conversion. These methods systematically affect key material properties like crystallinity, pore size and connectivity, surface area, metal oxidation states, ligand stability, and phase morphology. Consequently, these factors influence Zn^2+^ solvation/desolvation, ionic diffusion, electron transport, and the management of structural strain during repeated insertion and extraction in aqueous ZIBs. For instance, MOF-derived porous carbon combined with MnO_2_ nanoparticles exhibits stable Zn^2+^ storage, owing to enhanced conductivity and reduced Mn dissolution [10]. Similarly, MOF-coated Zn anodes utilize structured porous frameworks to balance Zn^2+^ flux and prevent dendrite formation via ionic-sieve functionality. Similarly, MOF-based interphases on Zn metal have been utilized to control Zn^2+^ flux and suppress dendrite growth through ionic-sieve behavior [11]. These synthetic and structural features underpin advanced design strategies for ZIB electrodes. Although other MOF families based on vanadium, nickel, iron, and multiple metals have been explored for aqueous zinc storage, we specifically focus on manganese-, copper-, and cobalt-centered systems for three main reasons. First, these metals form structurally durable MOFs and derivatives that can tolerate repeated Zn^2+^ insertion/extraction and water-related reactions under typical ZIB conditions. Second, Mn-, Cu-, and Co-MOFs are among the most extensively studied MOF-derived cathodes for ZIBS, enabling meaningful comparisons of their structures, processes, and performance metrics. Additionally, the relative abundance, affordability, and environmental friendliness of these transition metals make them more practical for widespread application than some more expensive or environmentally harmful alternatives. V-based MOFs and other innovative systems are briefly discussed in Section 3.4 as supplementary categories; however, they are not the primary focus of this comparison.

From a mechanistic standpoint, understanding how these MOF/MOF-derived materials store Zn^2+^ (and potentially H^+^ or OH^−^ in aqueous media) is crucial for advancing high-performance ZIBs. In situ and ex situ techniques (XRD, XPS, XAS, TEM), combined with electrochemical analysis, have uncovered various storage pathways: Zn^2+^ insertion into MOFs, conversion into metal oxides/hydroxides, co-insertion of Zn^2+^/H^+^, ligand-based redox processes, and dissolution or reprecipitation events in aqueous environments [2]. Additionally, a recent review underscored how MOFs and COFs are employed to stabilize Zn metal anodes by reducing dendrites, side reactions, and uneven Zn plating through porous framework interfaces [12]. Practically, achieving high capacity, excellent rate performance, and long-term cycling in full cells necessitates the integration of multi-scale design (from atomic active sites to nano-morphology and electrode architecture), water-stable conductive MOFs or durable derived composites, a thorough mechanistic understanding of metal/ligand roles, and the realization of large-format cells.

**Figure 1:**
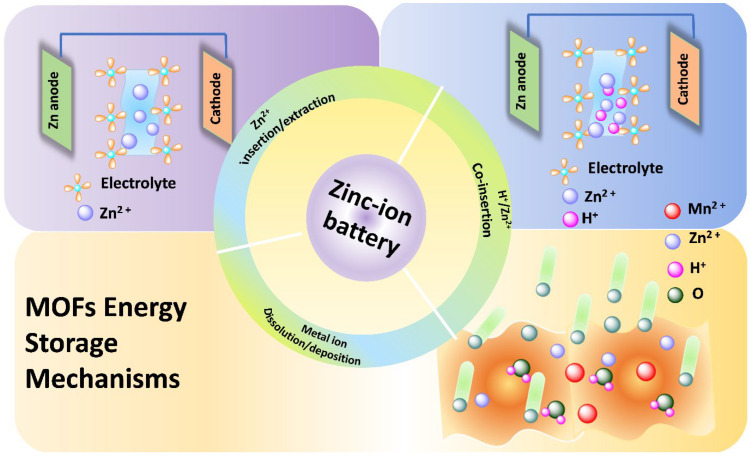
Schematic overview of recent advances in MOFs and MOF-derived materials for ZIBs, highlighting structure–property relationships, energy-storage mechanisms, and future development directions.

In summary, MOFs and MOF-derived materials based on Cu, Mn, Co and V serve as a versatile platform for the next generation of aqueous zinc-ion batteries. They offer various design options, including selecting metal centers, engineering linkers, modifying dimensionality, templated conversion, creating porous structures, and integrating composites to address challenges such as capacity, rate capability, and cycle life. By meticulously controlling the structure during synthesis, understanding mechanistic behavior, and aligning with electrode engineering, these MOF systems could accelerate the development of safe, affordable, high-performance zinc-ion batteries suitable for grid or stationary storage. The present paper provides an overview of current advancements in MOFs and MOF-derived materials for ZIBs, accompanied by a concise analysis of the impact of MOF structures on electrochemical behavior and energy-storage processes (Figure 1). Ultimately, we emphasize the principal problems that persist and delineate prospective avenues for the development of next-generation MOFs for high-performance ZIBs.

## 2. Synthesis Methods for MOFs

During the operation of ZIBs, Zn^2+^ ions dissolve from the anode and migrate to the cathode during discharge, where they are inserted into the crystal lattice of the cathode material. During charging, Zn^2+^ ions are extracted from the cathode and redeposited on the anode. This reversible Zn^2+^ insertion/extraction process is widely recognized as the fundamental energy-storage mechanism of ZIBs.

MOFs are composed of metal ions or clusters (as nodes) and organic ligands (as linkers), connected via coordination bonds, and represent a class of three-dimensional (3D) porous crystalline materials. Their open framework and exceptionally high surface area provide ideal channels and environments for Zn^2+^ intercalation and deintercalation. When Zn^2+^ ions enter the MOF pores, they interact with active sites within the framework, which may include open metal sites (e.g., Cu, Co, Mn) or oxygen- and nitrogen-containing functional groups (e.g., –COO^−^, –NH_2_) from the organic linkers. These interactions involve not only physical adsorption but also chemical bonding or electrostatic attraction, enabling the reversible insertion of Zn^2+^ ions. Electron transfer and energy exchange during this process collectively define the electrochemical energy-storage mechanism of ZIBs. Therefore, the structural design and chemical composition of MOFs play a crucial role in determining their electrochemical performance when used as electrode materials. MOFs are generally categorized into several representative structural types, including isoreticular metal–organic frameworks (IRMOFs), porous coordination networks (PCNs), and ZIFs. In addition, some MOFs are named after the research institutions or universities where they were first developed, such as MIL (Materials of Institut Lavoisier), UiO (University of Oslo), and HKUST (Hong Kong University of Science and Technology). By selecting appropriate metal ions (e.g., Fe^2+^, Co^2+^, Ni^2+^, Zn^2+^) and organic ligands (e.g., imidazole, carboxylic acids), the pore structure, electrochemical activity, and ion diffusion behavior of MOFs can be precisely tuned. In addition, constructing a rigid 3D framework helps maintain structural integrity during repeated charge–discharge cycles.

In summary, the selection of MOF type, structural modulation, and morphology optimization is critically important to enhancing their electrochemical performance in ZIBs. Rational design of metal nodes, ligands, and pore architectures via diverse synthetic routes can significantly enhance ion transport, electrical conductivity, and structural stability, thereby improving energy storage efficiency and cycling stability. The following section introduces four primary synthesis methods commonly used to prepare MOF materials.

### 2.1. Solvothermal Synthesis

Solvothermal synthesis is one of the most widely used and well-established methods for preparing MOFs. It involves the self-assembly of metal ions and organic ligands in an enclosed system under elevated temperature and pressure using organic solvents as the reaction medium, leading to the formation of crystalline MOF materials. By adjusting parameters such as solvent type, reaction temperature, duration, and precursor concentration, the crystal phase, pore size, particle size, and morphology of MOFs can be effectively controlled. This technique applies to a wide range of metal centers and ligands, typically yielding MOFs with high purity and productivity. The key to the solvothermal method lies in selecting an appropriate solvent system, controlling the temperature, and optimizing the metal ions/ligand ratio. For ZIBs cathodes, solvothermally synthesized MOFs can offer high porosity, robust frameworks, and tunable open metal sites (OMS), which significantly facilitate reversible Zn^2+^ insertion and charge transfer.

**Figure 2:**
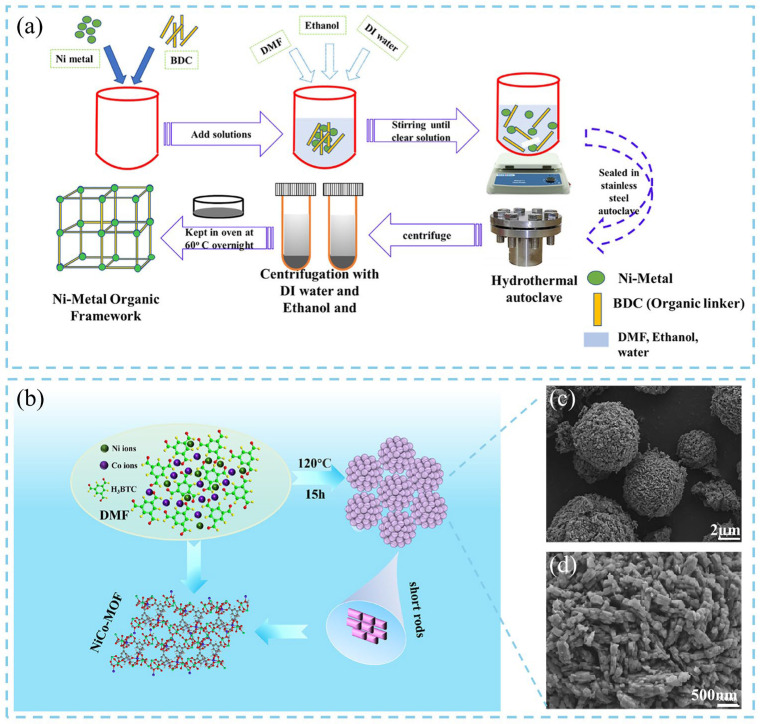
Schematic illustration of (**a**) the synthesis procedure of Ni-MOFs via one-pot solvothermal method [13], and (**b**) solvothermal synthesis of Ni, Co-MOF. (**c**,**d**) Morphological observation of Ni, Co-MOF-3 [15].

Ravipati et al. [13] synthesized a nickel-based MOF (Ni-MOF) via a one-pot solvothermal method, and the detailed synthesis process is illustrated in Figure 2a. Nickel nitrate hexahydrate (Ni(NO_3_)_2_·6H_2_O) and the organic linker terephthalic acid (BDC) were dissolved in a mixed solvent of DMF, ethanol, and deionized water with a specific volume ratio. The solution was magnetically stirred until completely homogeneous, forming the MOF precursor solution. The resulting mixture was then transferred into a 50 mL Teflon-lined stainless-steel autoclave and heated at 140 °C for 48 h. After the reaction, the autoclave was naturally cooled to room temperature, and the obtained precipitate was repeatedly washed with ethanol to remove residual impurities. Finally, the product was dried overnight at 70 °C in an oven to obtain purified Ni-MOF particles. Zheng et al. [14] investigated the effects of reaction temperature and duration parameters on the characteristics and electrochemical performance of Ni-MOFs synthesized via the solvothermal method. In their experiments, the same precursors were used to synthesize Ni-MOF samples at various temperatures (100–200 °C) and reaction durations (8–32 h). Field-emission scanning electron microscopy (FESEM) revealed that the Ni-MOFs consisted of hierarchical hollow architectures assembled from nanosheets. The average nanosheet size decreased with increasing temperature, while prolonged reaction time enhanced crystallinity. At low temperature (100 °C), the product yield was negligible due to high activation energy barriers, whereas at higher temperatures (200 °C), the MOF structure decomposed into corresponding metal oxides. At the optimized temperature of 160 °C, the product yield increased from 62.74% (8 h) to 73.04% (32 h), with negligible morphological change. Notably, the Ni-MOF synthesized under optimized conditions exhibited excellent electrochemical behavior, maintaining 118% of its initial capacity after 10,000 cycles in an aqueous electrolyte, indicating superior cycling stability. In general, bulk MOF materials exhibit poor electrical conductivity and limited structural stability, resulting in insufficient cycling life when used as electrode materials and thereby restricting their practical application in energy storage devices. In contrast, three-dimensional (3D)-MOFs exhibit larger surface areas, enhanced ion-transport pathways, and improved charge distribution. To further enhance MOF-based electrode performance, Zhang and co-workers [15] optimized the spatial architecture and particle size of bimetallic Ni, Co-MOFs synthesized via a solvothermal route. Recycled Ni(NO_3_)_2_·6H_2_O and Co(NO_3_)_2_·6H_2_O were used as metal sources, and by adjusting the Ni/Co molar ratios to 1:1, 1:2, 2:1, and 4:1, a series of NiCo-MOF-1/2/3/4 samples were obtained after reacting within DMF solvent at 120 °C for 12 h (Figure 2b). Morphological analyses revealed that all samples possessed spherical frameworks, but particle size and surface texture varied with metal composition. Higher magnification images showed that NiCo-MOF-1, -2, and -4 were composed of densely stacked layered structures, resulting in reduced surface areas and restricted ion diffusion. In contrast, NiCo-MOF-3 displayed a raspberry-like three-dimensional structure consisting of interconnected nanorods (Figure 2c,d), providing abundant pores and electrochemically active sites that facilitated redox reactions. Consequently, the NiCo-MOF-3 electrode retained 89.7% of its initial capacity after 7000 charge–discharge cycles, demonstrating outstanding stability and energy-storage performance.

### 2.2. Hydrothermal Synthesis

The primary distinction between hydrothermal and solvothermal synthesis refers to the type of solvent used and the associated chemical environment. Hydrothermal synthesis employs water as the solvent, offering advantages of operational simplicity, environmental friendliness, and low cost. Typically, when organic linkers containing functional groups (such as -COOH or -OH hydroxyl) or metal salts (e.g., Fe^3+^, Cr^3+^, Al^3+^, Zr^4+^, Ni^2+^, Co^2+^) are highly soluble in water, and the target MOF is insensitive to aqueous conditions, the hydrothermal method is preferred for MOF synthesis. Representative MOF examples, including MIL, MOF-74, UiO, and HKUST series, have been successfully synthesized via this approach.

**Figure 3:**
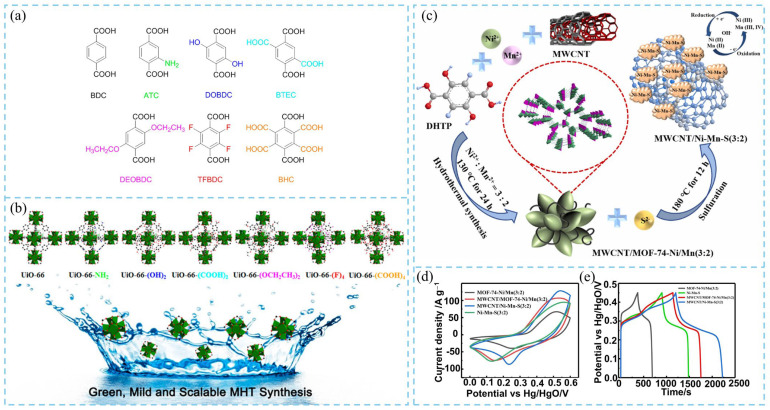
(**a**) BDC-type organic ligands used for the modulated hydrothermal synthesis (MHT) of UiO-66 MOFs. (**b**) Different UiO-66 MOFs obtained by the MHT method [16]. (**c**) Hydrothermal synthesis of MOF-74-Ni/Mn (3:2) and sulfurization process for preparing MWCNT/Ni–Mn–S (3:2) composite material. Electrochemical performance measurements for MWCNT/Ni-Mn-S composite electrodes. (**d**) CV curve at 10 mV s^−1^, and (**e**) GCD curve at 1 A g^−1^ of MWCNT/Ni–Mn–S [17].

Hu et al. [16] proposed a modulated hydrothermal synthesis (MHT) method for preparing water-stable UiO-66-type MOFs (UiO-66, UiO-66-NH_2_, UiO-66-(OH)_2_, and UiO-66-(COOH)_2_) using different organic linkers (Figure 3a). This strategy offers high reproducibility and scalability, enabling large-scale synthesis. Moreover, several new UiO-66 derivatives of UiO-66-(F)_4_, UiO-66-(OCH_2_CH_3_)_2_, and UiO-66-(COOH)_4_ were also developed using this method (Figure 3b). In a typical synthesis, approximately 5.2 mmol of Zr(NO_3_)_4_ and five mmol of different organic linkers were dispersed in aqueous solvents with varying ratios, and 70 equal (molar ratio) of acetic acid (AA) was introduced as a modulator, refluxing the reactions under heat for 24 h to obtain UiO-66-based MOFs. After washing and drying, crystalline products were obtained with yields ranging from 63% to 95%. XRD results showed that most samples exhibited the same topology as UiO-66. In contrast, UiO-66, UiO-66-(OCH_2_CH_3_)_2_, and UiO-66-(F) samples contained structural defects, which may be caused by the heterogeneity of organic ligands in aqueous solution. Notably, these defects could expose additional metal sites with Lewis-acidic character, which are beneficial for heterogeneous catalysis and electrochemical performance. FESEM images showed that MHT-synthesized samples consisted of quasi-spherical particles with diameters between 100 and 200 nm. The small particle size was attributed to rapid nucleation under reflux and the presence of modulators. Additionally, differences in linker polarity influenced molecular-level aggregation behavior in aqueous media, resulting in varied MOF morphologies. Thermogravimetric analysis (TGA) confirmed that all UiO-66-type MOFs synthesized via MHT maintained excellent thermal stability up to 400 °C, demonstrating strong potential for high-temperature applications. This work provides valuable guidance for the controlled, scalable synthesis of UiO-66-type MOFs and lays the foundation for their industrial production. In another study, Hao et al. [17] utilized the hydrothermal method to in situ grow bimetallic MOF-74-Ni/Mn (Ni/Mn molar ratio = 3:2) on the surface of multi-walled carbon nanotubes (MWCNTs), followed by sulfurization to obtain a 3D interconnected MWCNT/Ni–Mn–S (3:2) composite (Figure 3a). The unique interfacial contact and synergistic effects between the MOF-derived transition metal sulfides (TMSs) and the carbon framework markedly enhanced the electrochemical properties. This architecture facilitated fast electron/ion transport and alleviated volume expansion during long-term cycling, thereby improving electrode structural stability. Electrochemical measurements revealed that MWCNT/Ni–Mn–S (3:2) exhibited a larger CV area (Figure 3d) and longer discharge time (Figure 3e), indicating that MOF sulfurization and MWCNT incorporation significantly improved electrochemical performance. Consequently, the composite electrode maintained 93.3% capacitance retention after 20,000 cycles, demonstrating excellent energy density and cycling durability.

### 2.3. Ultrasonic-Assisted Synthesis Method

The ultrasonic-assisted synthesis method has been widely employed for the preparation of MOFs due to its cost-effectiveness, operational safety, environmental friendliness, and ability to drive reactions at relatively low temperatures. This technique relies on the formation and collapse of cavitation bubbles in the solvent, enabling efficient energy transfer and promoting chemical reactions. Studies have demonstrated that ultrasonic irradiation can induce new reaction pathways, facilitating the formation of materials with unique crystalline structures.

**Figure 4:**
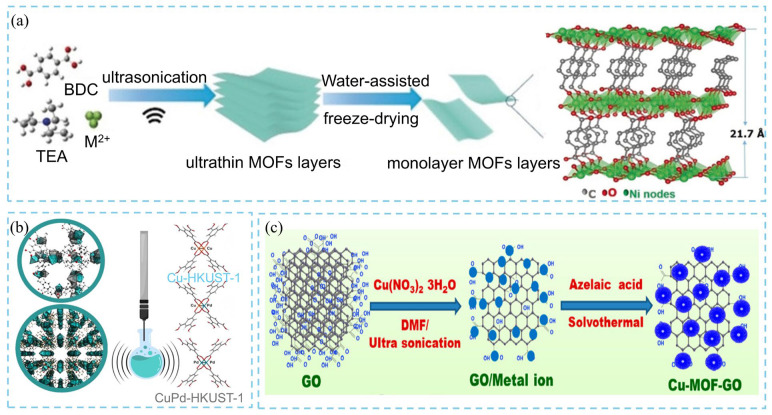
Schematic diagram of (**a**) ultrasonication-assisted synthesis of 2D Ni-MOF nanosheets [18], (**b**) structure of Cu-HKUST-1 and CuPd-HKUST-1 synthesized via ultrasound technique [20], and (**c**) ultrasound-assisted method for synthesis of size-controlled Cu-MOF-GO composite [21].

The synthesis of two-dimensional (2D) MOFs typically follows a bottom-up strategy, yielding ultrathin, well-dispersed layered structures via interfacial synthesis or surfactant-assisted methods. However, these conventional methods often suffer from long reaction times, complex procedures, substrate dependence, and low yields. In contrast, ultrasonic treatment can generate localized extreme conditions, with temperatures up to 5000 K and pressures up to 1000 bar, and rapid heating and cooling rates exceeding 10^10^ K·s^−1^. Such transient microenvironments provide favorable thermodynamic and kinetic conditions for the rapid nucleation and growth of MOF crystals. Han et al. [18] synthesized layered Ni-MOF via ultrasonic treatment and, by combining it with a water-assisted freezing process, successfully obtained ultrathin monolayer Ni-MOF nanosheets with a thickness of approximately 1 nm, as shown in Figure 4a. These works highlight that ultrasonic-assisted synthesis not only significantly shortens reaction time but also enables the controlled fabrication of diverse morphologies, including multilayer and monolayer 2D MOFs and MOF nanorods. This technique, therefore, provides an efficient and versatile route to the synthesis of MOF materials. Similarly, Du et al. [19] reported an ultrasonic-assisted bottom-up synthesis strategy for the direct preparation of uniform 2D Zn-BTC MOF nanosheets with an average thickness of 14 ± 2 nm. Some MOF-based composite nanomaterials are also synthesized via ultrasonic-assisted methods. Dymek and coworkers developed an ultrasound-assisted synthesis technique based on the HKUST-1 topology for the preparation of both monometallic and bimetallic MOF materials (Figure 4b) [20]. The results demonstrated that, under acidic conditions, the synergistic effect of ultrasonic and its unique reaction environment facilitates the simultaneous incorporation of two different metal cations into the MOF, leading to the successful synthesis of CuPd-HKUST-1 or CuPd-HKUST-1-son-def composites. This method allows effective regulation of the Cu/Pd ion ratio and distribution within the MOF structure. During the reaction, the ultrasonic technique not only enhances the catalytic effect of the acid and promotes the uniform dispersion of Pd species within the MOF but also significantly shortens the synthesis time. Finally, the researchers synthesized size-controlled copper-based MOF-decorated graphene oxide (GO) composites (Cu-MOF-GO) via ultrasound-assisted synthesis (Figure 4c) [21]. The results revealed that ultrasonic treatment of the GO–Cu^2+^ ion-mixed solution not only effectively reduced GO precipitation but also induced intense molecular collisions, significantly decreasing the GO colloidal particle size from 6.6 μm to 3 μm. Meanwhile, the ultrasonic exfoliation process successfully dispersed multilayer GO into single-layer GO sheets enriched with active functional sites. These reactive sites on the exfoliated GO layers facilitated the controlled nucleation and growth of small-sized MOF crystals, thereby maintaining uniform particle morphology.

### 2.4. Microwave-Assisted Synthesis

Microwave-assisted synthesis has been successfully applied to the preparation of various functional nanomaterials. Compared with conventional solvothermal methods, this approach directly heats the reactants through microwave irradiation. Microwaves are relatively low-energy electromagnetic waves with frequencies typically ranging from 0.2 to 300 GHz, enabling rapid energy transfer from electromagnetic radiation to reactant precursors and thereby accelerating chemical reactions. Moreover, microwave irradiation can promote the rotational alignment of specific molecules in the reaction mixture, thereby increasing molecular collisions and localized heating, further enhancing the reaction rate. Compared with hydrothermal or solvothermal techniques, microwave-assisted synthesis is safer, faster, and more energy-efficient, allowing for significantly shorter reaction times while maintaining high product yields. Due to these advantages, microwave synthesis has become one of the most widely adopted laboratory-scale methods for preparing MOF-based materials.

**Figure 5:**
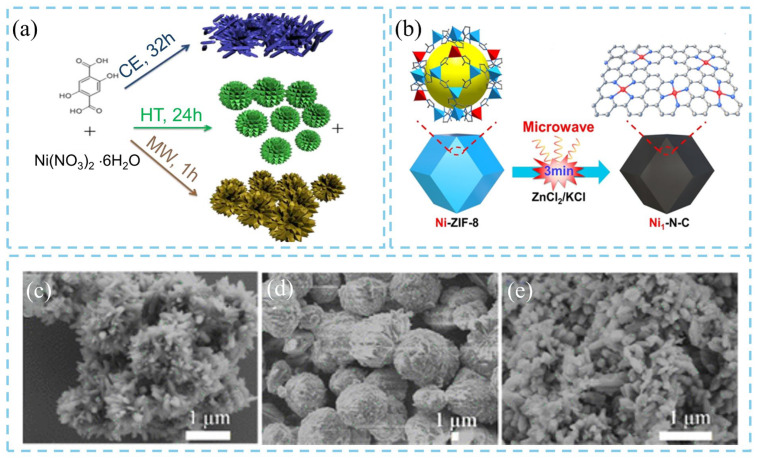
Schematic illustration of (**a**) MOF-74(Ni) synthesized through microwave-assisted synthesis (MW), heat treatment (HT), and capillary electrophoresis (CE) [22], (**b**) microwave-assisted rapid pyrolysis of Ni-MOF-8 for Ni-N-C preparation [24]. FESEM images of (**c**) MW-140, (**d**) HT-140, and (**e**) CE-140 [22].

The microwave-assisted synthesis has been reported to improve the pore uniformity and hydrophilicity of porous materials, thereby achieving unique characteristics that are difficult to achieve using traditional synthesis routes. For instance, Chen’s team synthesized MOF-74(Ni) materials using three different methods: microwave-assisted synthesis (MW), heat treatment (HT), and capillary electrophoresis (CE) (Figure 5a), and conducted a comparative analysis of their physicochemical properties and microstructures [22]. The results revealed that the microwave method is a rapid and reliable approach for producing high-quality MOF-74(Ni). FE-SEM images demonstrated significant morphological differences among the materials synthesized at 140 °C using different techniques. Specifically, the sample prepared via the microwave method (MW-140) exhibited a rice-grain-like morphology (Figure 5c), while those obtained from the heat-treatment (HT-140) and capillary electrophoresis (CE-140) methods displayed cauliflower-like (Figure 5d) and fragmented-grain (Figure 5e) morphologies, respectively. Notably, the MW-140 sample consisted of smaller and more uniformly distributed nanoparticles, which can be attributed to the higher nucleation rate induced by high-temperature microwave irradiation. Furthermore, extending the microwave treatment time from 5 min to 60 min resulted in more homogeneous, well-dispersed MOF-74(Ni) particles, indicating that a longer microwave reaction time promotes uniform crystal growth. Furthermore, the combination of ultrasonic and microwave strategies has also been developed for the fabrication of MOFs. During the process, ultrasonic treatment promoted extensive nucleation, while microwave irradiation accelerated the rapid growth of the MOF nanomaterials. Liu et al. [23] conducted both conventional solvothermal and microwave-assisted methods to synthesize Zr-fum-fcu-MOF. The effects of reaction temperature, time, and the amount of the modulator (formic acid, FA) on the product structure and yield were investigated. Research results demonstrated that microwave assistance had a minimal effect on the crystal structure or nucleation process of the MOF, but significantly enhanced the product yield (up to 83%) and reduced the reaction time to only 1 h under identical conditions. Additionally, researchers developed a versatile and straightforward microwave-assisted rapid pyrolysis method using a ZnCl_2_/KCl mixture as a microwave absorber. Taking Ni-doped zeolitic imidazolate framework-8 (Ni-ZIF-8) as the precursor, single-atom Ni embedded in N-doped carbon (Ni–N–C) was obtained within 3 min of microwave treatment without the need for an inert atmosphere (Figure 5b) [24]. This process markedly improved both preparation efficiency and energy utilization. Finally, Fan et al. [25] applied the ultrasonic–microwave method to fabricate UiO-66 membrane on an aluminum oxide (AAO) substrate. Due to the synergistic effects of ultrasound-induced nucleation and microwave-driven growth, a high-quality ultrathin polycrystalline UiO-66 membrane with a thickness of approximately 210 nm was successfully fabricated within 1 h, whereas the conventional solvothermal method required 24 h at 130 °C to achieve comparable results. Therefore, this synergistic strategy, which combines ultrasonic nucleation with microwave-assisted rapid heating, can significantly enhance the synthesis efficiency and structural controllability of MOF materials. It provides a new, scalable route to controlled morphological construction with diverse precursor systems, demonstrating great potential for large-scale production and practical applications.

## 3. MOFs and MOF Derivatives for Zn-Ion Batteries

Pristine metal–organic frameworks such as Prussian blue and its analogs (PB/PBAs), as well as manganese- and vanadium-based frameworks and conductive two-dimensional frameworks, have emerged as promising cathode materials for aqueous zinc-ion batteries. Their open structures facilitate zinc ion diffusion, their redox-active metal centers can be adjusted, and they are straightforward to synthesize. PBAs are especially notable for their rigid, open framework and their capacity to reversibly insert and remove Zn^2+^ ions, which boosts their rate capability and scalability, particularly when their structural defects and water content are optimized [26]. Besides PBAs, Mn- and V-based MOFs offer alternative redox couples and frequently exhibit higher theoretical capacities. However, they usually require compositional modifications or structural stabilization to prevent dissolution or failure during cycling [27]. Conductive MOFs and frameworks with integrated electronic pathways reduce the need for excessive conductive additives and enhance rate performance by addressing the low electronic conductivity common in many pristine MOFs. Recent reviews thoroughly discuss these design strategies and their mechanisms within the context of MOF-based materials for aqueous ZIBs.

MOF-derived materials expand the uses of MOFs by acting as templates or precursors to produce porous carbons, metal or metal-oxide (or sulfide) nanoparticles within carbon matrices, and hierarchical composites. These improvements enhance electrical conductivity, active-site accessibility, and mechanical durability against volume expansion. When Zn-MOFs or ZIF precursors undergo carbonization, they generate high-surface-area porous carbons that buffer structural strain, enable ion transport, and host uniformly distributed active species. Converting MOF oxides or sulfides, such as those derived from V and Mn, often yields nanostructured cathodes with more active sites and faster kinetics. Coatings from MOFs and MOF-based interlayers are also used to stabilize Zn anodes, reduce dendrite growth, and prevent corrosion. This demonstrates MOFs’ dual role as cathode precursors and as functional interface modifiers in electrolytes or anodes. Recent reviews summaries these strategies and highlight that understanding the microstructure of synthesis products and Zn storage mechanisms is essential for advancing practical MOF-based Zn-ion batteries.

### 3.1. Mn-MOFs and Their Derivatives for Zn-Ion Batteries

Recently, Mn-based MOFs have gained significant interest as cathode hosts in liquid zinc-ion batteries (ZIBs). Their porous structures are adaptable, contain redox-active Mn centers, and can incorporate or modify Zn^+^. For example, a study with Mn(BTC) (Mn-benzene-1,3,5-tricarboxylate) MOF showed it could store approximately 112 mAh g^−2^ in a ZIB. Zn^+^ storage involved the MOF converting to MnO_2_ during cycling [28]. The researchers attributed this to the ordered pore design, which promotes Zn^+^ diffusion, and the Mn redox centers, which facilitate charge storage. Recently, a microporous Mn-MOF (Mn-BTC with an octahedral shape) was used as a cathode in water-based ZIBs, achieving approximately 119 mAh g^−3^ after 1000 cycles at 500 mA g^−3^, demonstrating good stability [9]. These results indicate that although pure Mn-MOFs face challenges in electrical conductivity and cycle stability, they provide valuable insights into Zn^2+^ storage mechanisms and potential improvements to ZIB cathode design.

Beyond pure MOFs, materials derived from Mn-MOFs, such as MnOx embedded in carbon or hierarchical Mn_2_O_3_@C made from Mn-MOF templates, perform better by addressing common issues, including poor conductivity and limited active-site exposure. For example, Mn_2_O_3_@C flakes from a Mn-MIL-100 precursor achieved a bulk capacity of about 154.9 mAh cm^−3^ and maintained roughly 79.6% of their capacity after 3000 cycles in a fiber-shaped ZIB device [29]. Additionally, a MOF-templated porous MnOx@N-C cathode delivered approximately 305 mAh g^−3^ after 600 cycles at 500 mA g^−1^, demonstrating the benefits of combining MOF-derived nanostructures with a conductive carbon matrix [1]. The core idea is that MOFs contain evenly distributed precursors and tunable pores. When subjected to controlled pyrolysis or conversion, they produce uniformly dispersed Mn oxide nanoparticles within a carbon scaffold, enhancing electronic pathways, maintaining structural integrity during Zn^+^ intercalation or conversion, and minimizing Mn^+^ dissolution. Adding Mn^2+^ to the electrolyte can also prevent Mn dissolution and improve cycle stability in Mn-MOF-based cathodes [28]. Overall, Mn-MOFs and their derivatives are promising for scalable, high-stability water-based ZIB cathodes. However, further research is needed to address challenges such as permeability, active material utilization, and contact stability.

**Figure 6:**
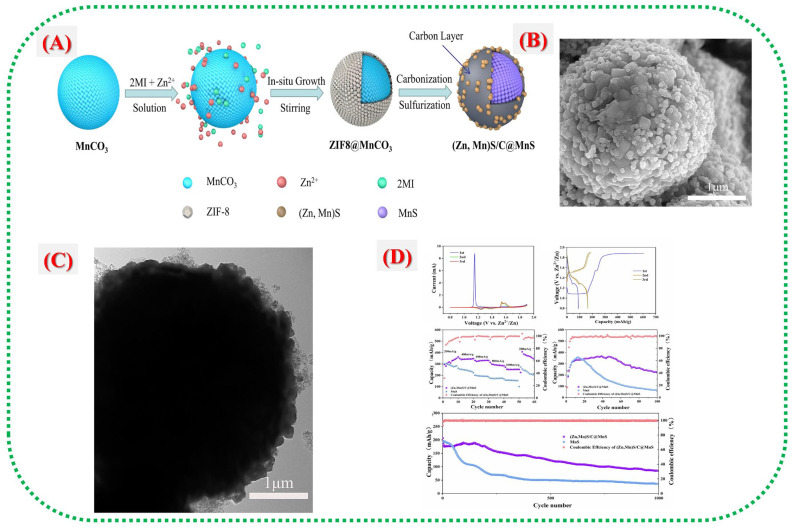
(**A**) Schematic illustration of the (Zn, Mn)S/C@MnS composites. (**B**) SEM image of the (Zn, Mn)S/C@MnS composite. (**C**) TEM image of the (Zn, Mn)S/C@MnS composite. (**D**) CV curves of the (Zn, Mn)S/C@MnS cathode at a scan rate of 0.2 mV s^−1^, discharge/charge profiles of the (Zn, Mn)S/C@MnS cathode during the first three cycles at 200 mA g^−1^, rate performance of the (Zn,Mn)S/C@MnS and MnS cathodes at various current densities, cycling performance of the (Zn, Mn)S/C@MnS and MnS cathodes at 200 mA g^−1^, cycling performance of the (Zn, Mn)S/C@MnS and MnS cathodes at a current density of 1000 mA g^−1^ [30].

Manganese-based metal–organic frameworks (Mn-MOFs) and their derivatives offer a promising approach to address common issues with traditional Mn-based cathodes in water-based Zn-ion batteries (ZIBs), including poor conductivity, dissolution, and slow ion transport. MOFs can be converted into functional composites, such as sulfides, oxides, and carbon-coated hybrids. This method enables the development of well-structured, porous architectures and conductive networks that enhance ion transport while maintaining structural stability during battery cycling. Wang et al. [30] developed a heterostructured microsphere composed of (Zn, Mn)S/C-derived MOF coated with MnS ((Zn, Mn)S/C@MnS) to improve the performance of MnS cathodes. The process, shown in Figure 6A), begins with synthesizing MnCO_0_@ZIF-8 microspheres, followed by carbonization and sulphuration to form a (Zn, Mn)S/C shell around a MnS core. The in situ growth of ZIF-8 and its subsequent conversion into a conductive carbon layer help prevent MnS dissolution and enhance electrical conductivity. Figure 6B,C depict the microstructural evolution from smooth ZIF-8@MnCO_3_ seeds to rough (Zn, Mn)S/C@MnS microspheres, indicating successful transformation and hollow-shell formation that facilitate Zn^+^ diffusion. Electrochemical tests in Figure 6D reveal that this composite surpasses other materials, achieving a high specific capacity of 374.1 mAh g^−1^ at 0.2 A g^−1^ and maintaining 85.6 mAh g^−1^ after 1000 cycles at 1 A g^−1^. These results demonstrate that the MOF-derived carbon layer and heterostructure (Zn, Mn)S interfaces work together to enhance redox reversibility, prevent Mn dissolution, and preserve cathode structural stability during prolonged cycling.

**Figure 7:**
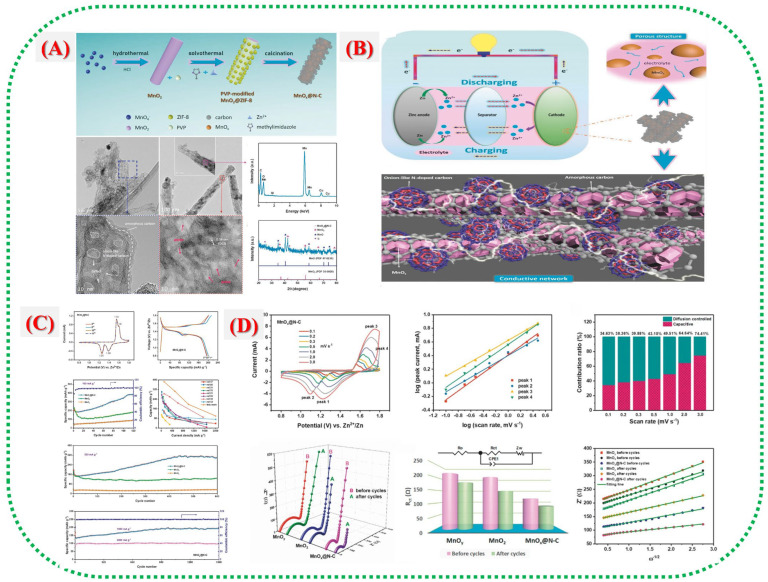
(**A**) shows a schematic illustration of the MnOx@N-C composite, along with its TEM images and XRD pattern (including MnO and MnO_2_ standards for comparison). (**B**) depicts a schematic of a ZIB with a Zn anode and MnOx@N-C cathode, highlighting the enlarged cathode structure, internal porous MnOx nanoparticles for improved ion transport, and the interconnected N-doped carbon network. (**C**) presents CV curves of the MnOx@N-C electrode at 0.1 mV s^−1^ after various cycles; discharge/charge profiles; cycling performance of MnOx@N-C, MnO_2_, and MnOy electrodes at 100 mA g^−1^; long-term cycling at 500 mA g^−1^; high-rate cycling of MnOx@N-C; and a comparison of stable capacities with other Mn-based ZIB materials. (**D**) shows CV curves of MnOx@N-C at different scan rates; log(i)–log(v) analysis; capacitive and diffusion-controlled contribution ratios; Nyquist plots of MnOx@N-C, MnO_2_, and MnOy before and after cycling; a comparison of Rct values; and Z′ vs. ω^−1/2^ linear relationships in the low-frequency region [1].

Fu et al. [1] introduced a new porous MnOx@N-doped carbon nanorod composite made from a ZIF-8 template. This work demonstrates that MOF-derived heterostructures can significantly enhance Zn-ion storage. As shown in Figure 7A,B, the process starts with wrapping MnO_2_ nanorods with ZIF-8, followed by high-temperature carbonization that forms onion-like layers of N-doped carbon around the porous MnOx nanorods. TEM images (Figure 7A) reveal that the nanorod shape remains intact, with numerous mesopores and circular graphitic carbon layers that enhance electron transport and structural stability. XPS patterns in Figure 7 indicate the coexistence of Mn^2+^ and Mn^3+^ states, suggesting the presence of mixed-valence Mn species capable of reversible redox reactions. Nitrogen species, such as pyridinic, pyrrolic, and graphitic N, derived from ZIF-8 provide active sites and improve electrical conductivity. Electrochemical tests (Figure 7C,D) show a high reversible capacity of 305 mAh g^−1^ at 0.5 A g^−1^ and excellent stability over 1600 cycles, outperforming most Mn-based ZIB cathodes. The N-doped carbon network fosters electron flow and prevents degradation of the MnOx core. Synchrotron-based XANES and ex situ XRD studies (Figure 7A) further confirm the reversibility of the structure and the rapid reaction rates enabled by the MOF-based design, allowing for reversible redox between Mn^5+^ and Mn^2+^ during charge and discharge. These findings underscore that MOF templates serve not only as building blocks but also as nitrogen-doped carbon sources, ensuring high conductivity and electrochemical durability.

**Figure 8:**
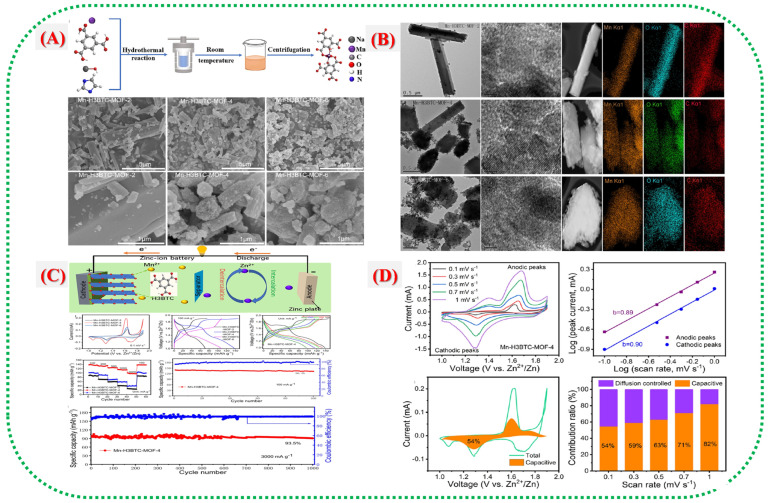
(**A**) schematic of the synthesis route and Mn(II) coordination in Mn-H_3_BTC-MOF-4, with SEM images of Mn-H_3_BTC-MOF-2, -4, and -6. (**B**) HRTEM images and EDS elemental maps (C, O, Mn) of Mn-H_3_BTC-MOF-2, -4, and -6 powders. (**C**) ZIB schematic using MOF cathode and Zn anode; CV curves at 0.1 mV s^−1^; charge/discharge profiles at 100 mA g^−1^; rate capability and multi-current performance of Mn-H_3_BTC-MOF-4. (**D**) CV curves of Mn-H_3_BTC-MOF-4 at different scan rates; log(i)–log(v) plots; capacitive versus diffusion contributions; bar chart of capacitive ratios at various scan rates [31].

**Figure 9:**
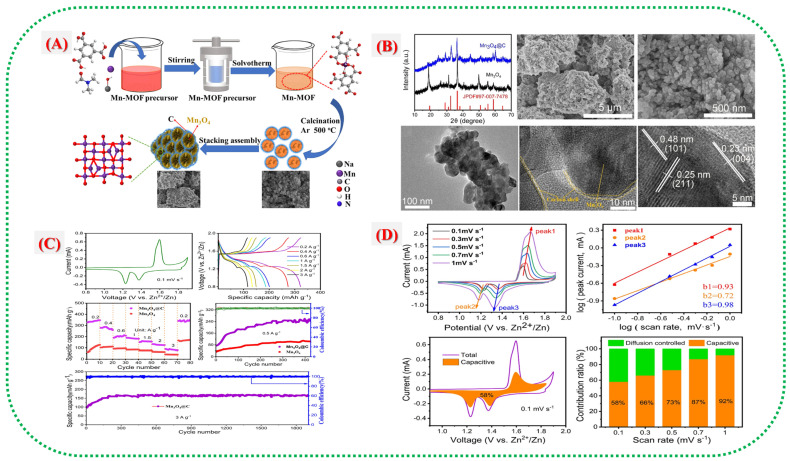
(**A**) Schematic illustration of the synthesis process and atomic-scale formation mechanism of Mn_3_O_4_@C nanospheres. (**B**) Characterization of Mn_3_O_4_@C: XRD, SEM, TEM, and HRTEM analyses. (**C**) Electrochemical performance of Mn_3_O_4_@C: CV at 0.1 mV s^−1^, GCD at various current densities, rate capability, and cycling performance at 0.5 and 3 A g^−1^. (**D**) CV curves of Mn_3_O_4_@C at different scan rates, log(v)–log(i) fitting, capacitive contribution analysis at 0.1 mV s^−1^, and capacitive contribution diagram [31].

Yin et al. [31] demonstrated how Mn–MOFs can be directly used as active cathode materials. They investigated coordinatively unsaturated Mn–MOFs that enable rapid Zn^+^ intercalation and showed excellent performance. The study introduces three variants of Mn-H_3_BTC-MOF: Mn-H_3_BTC-MOF-2, -4, and -6, each featuring different sites where Mn^2+^ ions interact with carboxylate ligands. As shown in Figure 8A, varying the Mn-to-ligand ratio affects the level of coordination unsaturation, thereby influencing electron and ion transport within the material. SEM and TEM images (Figure 8A,B) reveal that the Mn-H_0_BTC-MOF-4 sample is highly porous, uniformly shaped, with particles around 500 nm and more open channels suitable for Zn^+^ ion flow. The electrochemical profiles in Figure 8C,D display distinct redox peaks associated with bidirectional Mn^2+^/Mn^3+^ transitions. Among the three samples, Mn-H_0_BTC-MOF-4 exhibits the highest capacity (138 mAh g^−1^ at 0.1 A g^−1^) and excellent cycle stability (93.5% capacity retention after 1000 cycles at 3 A g^−1^). This superior performance stems from an optimal level of coordination unsaturation, which enhances activity by promoting the mobility of electrons and Zn^+^ ions. Figure 9 illustrates the long-term cycling and rate performance, highlighting its durability and rapid response. These findings present a new approach, adjusting coordination unsaturation to develop highly active and stable Mn-MOFS for water zinc-ion batteries, combining molecular-level control with improved electrochemical performance.

Yin et al. developed a new Mn–MOF composite system (Figure 8A–D) that combines manganese oxide with carbon [31]. These hybrids capitalize on the inherent redox activity of Mn centers and the electrical conductivity provided by the carbon frameworks. Figure 8A depicts a schematic of the transformation of a MOF into a hybrid via solvothermal and carbonization methods. The structural insights in Figure 8B reveal that solid Mn–O phases form within the amorphous carbon network. Electrochemical testing in Figure 8C,D indicates stable charge–discharge cycles, low resistance, and strong rate capability. These promising results arise from the combined benefits of the MOF-derived porous architecture and the conductive carbon matrix. This interconnected framework helps prevent Mn degradation, absorbs structural stress during Zn^+^ intercalation and deintercalation, and improves cycle stability.

Figure 9A illustrates the process of synthesizing the Cu/MnO@C hybrid from a Cu–Mn bimetallic MOF (Cu/Mn–BTC), as described by Wang et al. The MOF was prepared via static layer-by-layer self-assembly and then heated to form a Cu/MnO@C hybrid with an embedded three-dimensional carbon framework. This process preserved the MOF’s porous structure, generated oxygen vacancies, and ensured uniform dispersion of Cu and Mn species, thereby creating multiple electrochemically active sites. The carbon shell coating prevents Mn dissolution and enhances the structural stability during repeated Zn^+^ ion insertion and extraction. Figure 9B illustrates the physical and microstructural features that confirm the formation of Cu/MnO nanoparticles encapsulated by carbon layers. When copper is added, the microspheres become smaller and denser, thereby enhancing electrical conductivity and reaction kinetics. HRTEM and HAADF–TEM analyses reveal lattice edges consistent with MnO (111) and CuO (110) planes, along with uniform elemental distribution, indicating successful Cu integration into the MnO matrix. The electrochemical performance data (Figure 9C,D) show that the Cu/MnO@C electrode has a significantly higher specific capacity (311.5 mAh g^−1^ at 0.1 A g^−1^) than pure Mno@c. It also demonstrates excellent cycling stability, maintaining 114.3 mAh g^−1^ after 500 cycles at 1 A g^−1^, with an 88% retention. Cyclic voltammetry reveals smaller potential gaps and an additional redox peak at approximately 0.96 V, indicating a Cu^+^/Cu^0^ redox process. A comprehensive overview of recent literature on Mn-MOFs and their derivatives is presented in Table 1. The hybrid energy-storage mechanism in Figure 7 involves reactions that combine H^+^/Zn^2+^ intercalation and extraction with Cu^2+^/Cu^0^ conversion. By integrating intercalation pseudocapacitance and conversion mechanisms, this system provides enhanced capacity, rate performance, and durability.

Overall, these studies demonstrate that Mn–MOFs and their variants play a vital role in enhancing the performance of Zn-ion battery cathodes. Due to MOFs’ unique coordination chemistry, adjustable porosity, and structural flexibility, they enable precise control over electronic structures and ion transport pathways. Carbon layers derived from MOFs efficiently conduct electricity and protect Mn-based active sites from solution degradation. Simultaneously, combining heterostructures such as (Zn, Mn) S surfaces, N-doping, or creating coordination unsaturation significantly increases redox activity and charge-transfer efficiency. Consequently, Mn–MOF-based alloys attain very high specific capacities (around 370 mAh g^−1^), excellent rate performance, and stable cycling exceeding 1000 cycles, outperforming most MnO_2_ or MnS cathodes. These findings lay the foundation for future MOF-based electrode development, emphasizing atomic-level precision, heterostructure integration, and conductive network design as essential strategies for next-generation high-performance water Zn-ion batteries.

### 3.2. Cu-MOFs and Their Derivatives for Zn-Ion Batteries

Using Cu-MOF-derived materials as cathodes in ZIBS addresses common problems in traditional vanadium- and manganese-based systems, such as poor conductivity, dissolution, and slow reaction kinetics. Transforming Cu–MOFs into mixed-metal frameworks, multifunctional oxides, and phosphates creates unique morphologies, larger surface areas, and enhanced redox activity, all of which are essential for improving cycle stability and Zn^2+^ storage capacity.

**Figure 10:**
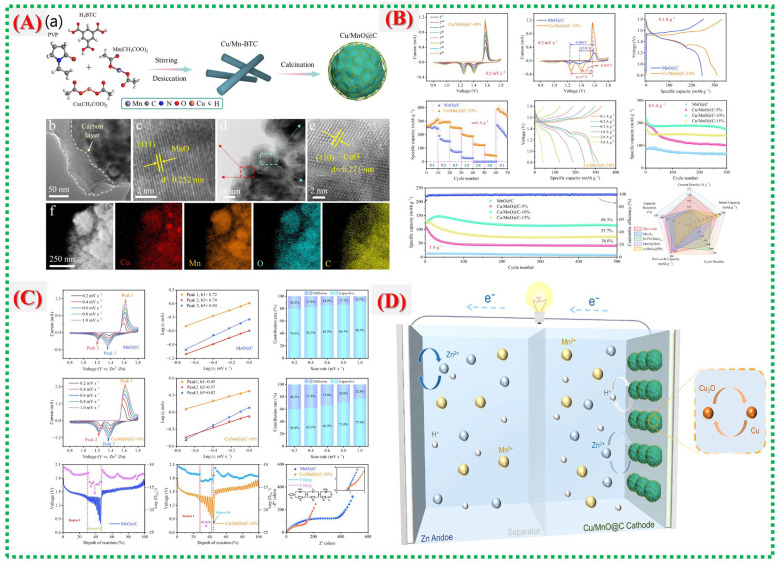
(**A**) (**a**) Schematic of Cu/Mn–MOF transformation into Cu/MnO@C with carbon coating and oxygen vacancies. (**b**–**f**) TEM, HRTEM and EDS images of Cu/Mn–MOF. (**B**) CV showing hierarchical microspheres and stable, enhanced redox peaks after Cu doping. (**C**) XRD, Raman, and XPS confirm MnO phase, Cu incorporation, carbon defects, and mixed-valence states. (**D**) Electrochemical performance showing high capacity and excellent cycling stability for Cu/MnO@C-10% [39].

Lu et al. developed a Cu–MOF-derived Cu–MnO@C composite as a high-performance cathode for aqueous Zn-ion batteries, showing that Cu doping, carbon coating, and oxygen vacancy engineering collectively enhance conductivity, stability, and redox kinetics [39]. Their research confirmed that MOF-based methods create a porous structure that significantly improves long-term cycling and Zn^2+^ intercalation. They detailed the synthesis process in Figure 10A, in which Cu/Mn–MOF precursors undergo static deposition and calcination to produce the composite. In this process, organic ligands break down into conductive carbon, oxygen vacancies form, and Mn^2+^ is partially replaced by Cu^2+^. The schematic illustrates how the MOF template enhances electrochemical performance by enabling Cu integration into the MnO lattice while preserving porosity.

Figure 10A(b–f) illustrates the morphological development and electrochemical activation behavior. Cu/MnO@C consistently forms micro-nano-hierarchical spheres, especially in the optimal Cu/MnO@C-10% composition, as shown in SEM images. CV curves display redox peaks associated with the Mn^2+^/Mn^3+^ and Cu^2+^/Cu^+^ transitions, indicating that Cu doping increases the number of active redox centers, reduces polarization, and accelerates charge-transfer kinetics. The overlapping CV curves in later cycles demonstrate the response’s steady reversibility (Figure 10B,C). These findings confirm that the composite is a defect-rich, carbon-coated, mixed-valence structure suitable for electrochemical applications. Finally, Figure 10C,D illustrates the electrochemical performance, with Cu/MnO@C-10% achieving a high capacity of 311.5 mAh g^−1^ at 0.1 A g^−1^ and retaining 88.3% after 500 cycles. Doping, defect engineering, and carbon encapsulation work together to enhance charge transfer and structural stability during extended cycling, thereby accounting for this outstanding performance.

**Figure 11:**
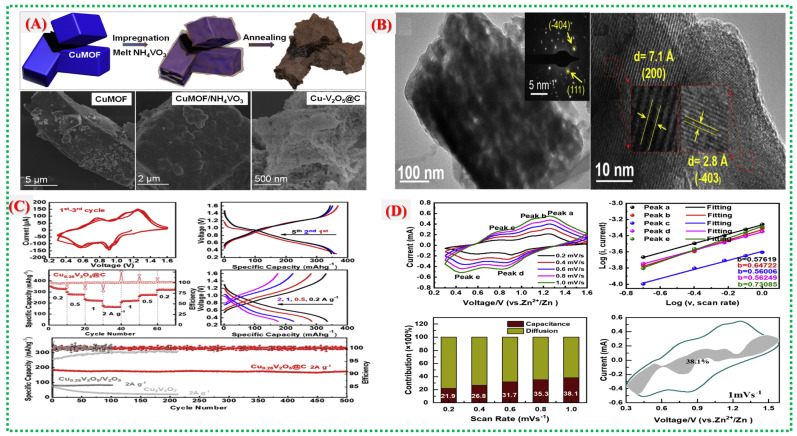
(**A**) Schematic of phosphate-assisted transformation of Cu–MOF to Cu_2_(OH)PO_4_ nanosheets, SEM images of Cu-MOFs. (**B**) TEM images showing ultrathin porous nanosheets supported on a carbon network. (**C**) Electrochemical performance of Cu_0.26_V_2_O_5_@C: initial CV curves at 0.2 mV s^−1^; charge–discharge profiles at 0.2 A g^−1^; rate capability; corresponding charge–discharge curves; and cycling stability compared with Cu_0.26_V_2_O_5_/V_2_O_5_ and Cu_2_V_2_O_7_ at high current densities. (**D**) Kinetic analysis of Cu_0.26_V_2_O_5_@C:CV curves at various scan rates; log(i)–log(v) plots of redox peaks; capacity contribution at different scan rates; and pseudocapacitive contribution to the CV response at 1.0 mV s^−1^ [40].

Wang et al. [40] reported a Cu_2_(OH)PO_4_ nanosheet material derived from a Cu-MOF, anchored to a carbon framework, for use in Zn-ion batteries. They showed that phosphate-assisted transformation of the MOF precursor produces ultrathin nanosheets with many defects and hierarchical porosity. Their study demonstrated that combining porous nanosheets with a conductive carbon network enables rapid electron transfer, efficient Zn^2+^ intercalation, and excellent cycle stability. Using the MOF as a structural template, Wang et al. described how phosphate induces the conversion of the Cu–MOF precursor into Cu_2_(OH)PO_4_, as illustrated in Figure 11A. The porous MOF skeleton ensures uniform ion transport, and the controlled transformation results in ultrathin nanosheets rich in edge sites. This schematic highlights the preservation of structural porosity and the formation of a nanoscale framework ideally suited for high-rate Zn-ion storage.

Figure 11B presents SEM and TEM images illustrating ultrathin Cu(OH)PO_4_ nanosheets supported on a conductive carbon matrix. These nanosheets feature numerous surface imperfections and interconnected holes, facilitating rapid Zn^2+^ ion transport and efficient electrolyte infiltration. The short ion diffusion pathways and high surface area enhance charge transfer kinetics and lower internal resistance. Figure 11C displays detailed electrochemical analyses, including CV, GCD, and kinetic assessments. The GCD curves exhibit stable charge–discharge plateaus aligned with redox signatures, while the CV plots reveal multiple reversible redox transitions involving Cu^2+^/Cu^+^. Kinetic studies indicate that the material exhibits a mixed diffusion–capacitive behavior, with vigorous pseudocapacitive activity and swift charge storage, as supported by b-value analysis and capacitive contributions. Rate capability and long-term cycling stability are demonstrated in Figure 11D, with the electrode retaining over 2000 cycles and delivering more than 300 mAh g^−1^ at 0.2 A g^−1^. The carbon framework maintains consistent electrical conductivity, and the nanosheet structure accommodates volume changes, preventing disintegration and ensuring durability.

**Figure 12:**
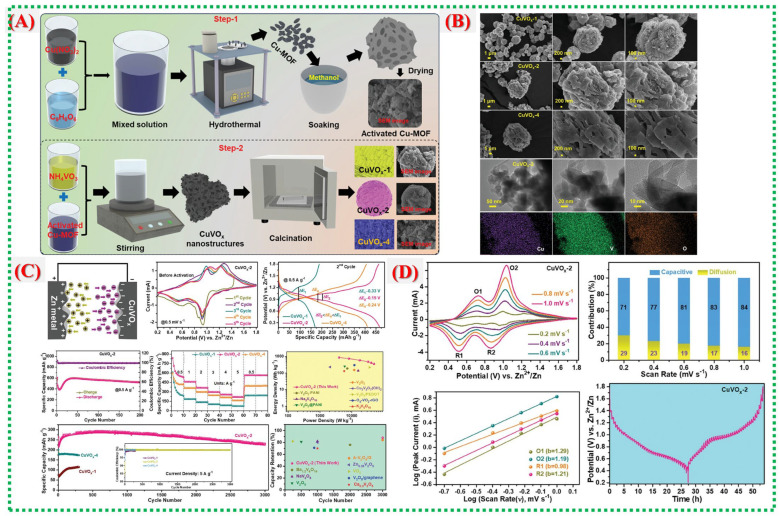
(**A**) Schematic of dissolution–regrowth formation of CuVO_x_ from Cu–MOF precursors. (**B**) SEM/TEM showing morphology evolution and optimized porous CuVO_x_-2 network. (**C**) XRD/Raman/XPS confirming mixed Cu-V oxide phases and multiple oxidation states. (**D**) Electrochemical performance showing high capacity, strong rate capability, and stable cycling [41].

Kakarla et al. synthesized Cu–MOF-derived CuVO_x_ nanostructures with tunable compositions for efficient Zn-ion storage [41]. Their study shows that shape, phase, and redox activity can be adjusted by varying the proportion of vanadium precursor. The best-performing CuVO_x_-2 sample features a hierarchical porous network and synergistic Cu–V redox interactions, providing high capacity and stability. When vanadium is added, the Cu–MOF template partially dissolves, following the dissolution–regrowth scheme illustrated in Figure 12A by Kakarla et al. The concentration ratio influences the shape of the regenerated CuVO_x_ materials. Ultimately, this process produces a hierarchical porous structure with evenly distributed Cu–V–O domains that facilitate fast electron transport and Zn^2+^ diffusion. SEM and TEM images in Figure 12B illustrate the morphological evolution: from scattered nanoparticles in CuVO_x_-1 to interconnected porous frameworks in CuVO_x_-2, and then to clustered agglomerates in CuVO_x_-4 as vanadium concentration grows. Thanks to its balanced porosity and uniform structure, the CuVO_x_-2 sample enhances active-site utilization and promotes rapid ion mobility.

Elemental mapping and BET analysis reveal mesoporosity and a homogeneous elemental distribution, both of which are essential for fast Zn^2+^ intercalation and capacity retention. Figure 12C,D displays the electrochemical performance, demonstrating that the CuVO_x_-2 electrode delivers a high specific capacity exceeding 500 mAh g^−1^ at 0.5 A g^−1^ and maintains 220 mAh g^−1^ at 5 A g^−1^. Cycling tests confirm excellent structural stability with minimal performance degradation. The superior performance arises from the synergistic redox effects of Cu and V, abundant active interfaces, and the well-developed porous structure derived from the MOF template. Table 2 summarizes recent studies on Cu-MOFs and their derived materials.

These three studies collectively highlight the importance of surface functionalization, hybrid composite development, and MOF-derived structures in significantly enhancing Zn-ion storage capabilities. They demonstrate that creating high-capacity, fast, and durable Zn-ion batteries is achievable through modifications in precursor chemistry, controlling the crystalline and morphological transformations of MOF-derived metal oxides, and incorporating conductive or active surface groups. The primary conclusion is that improving electrochemical performance, aiming for high energy density, long cycle life, and excellent rate performance, requires precise atomic-scale modifications, such as heteroatom doping, micro- and nanoscale structuring, and optimization of ion/electron transport pathways.

### 3.3. Co-MOFs and Their Derivatives for Zn-Ion Batteries

**Figure 13:**
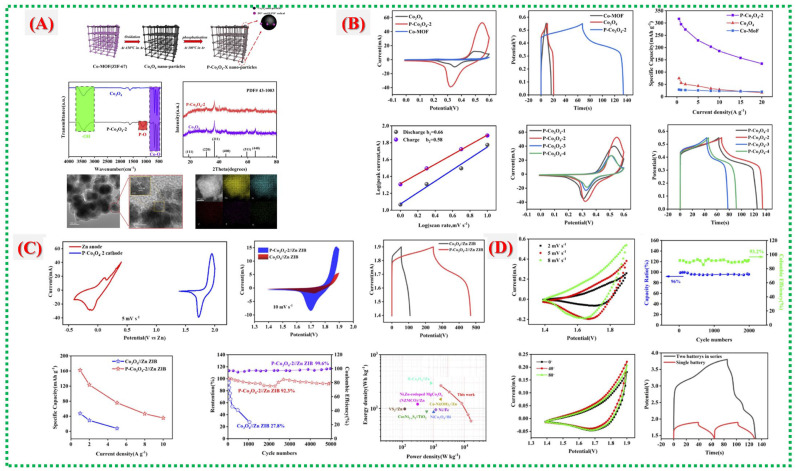
(**A**) Synthesis, schematic and structural characterization of PCO (FT-IR, XRD, TEM/HRTEM, HAADF-STEM/EDS). (**B**) Electrochemical performance of PCO samples (CV, GCD, rate capability, and kinetics). (**C**) Comparison of PCO-2||Zn ZIB with Co_3_O_4_||Zn ZIB (CV, GCD, rate performance, cycling, Ragone plot). (**D**) Electrochemical performance of flexible all-solid-state fiber-shaped Zn–Co battery (CV, cycling stability, bending tests, and series connection) [54].

Pan Xiao et al. conducted a detailed structural and electrochemical study of phosphorus-functionalized Co_3_O_4_ (PCO) derived from Co-MOF precursors, as reported in this paper [54]. They established a link between Zn-ion storage behavior, surface chemistry, and MOF-derived morphology. The synthesis process and structural analysis are shown in Figure 13A. TEM and elemental mapping reveal lattice expansion and uniform elemental distribution, enhancing Zn^2+^ diffusion and surface activation. Electrochemical performances of all PCO variants, displayed in Figure 13B, show that PCO-2 has the largest CV area, the longest discharge plateau, the highest capacity, and favorable diffusion-controlled kinetics- all indicating improved redox activity. Faster charge-transfer and surface reactions in PCO-2 enable the Zn-ion battery (PCO-2||Zn) to reach higher capacity and power, maintaining high-rate stability, as shown in Figure 13C. Reduced impedance and increased reversibility, demonstrated in Figure 13D, indicate that phosphorus functionalization alters electron density, lowers the band gap, and accelerates OH^−^ adsorption, collectively leading to high-performance Zn-ion storage.

**Figure 14:**
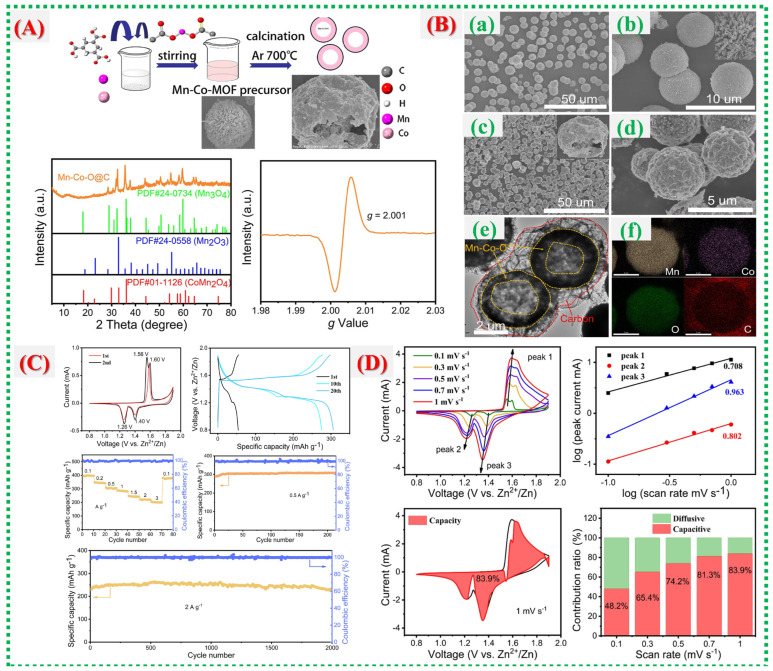
(**A**) Synthesis schematic of hollow Mn–Co–O@C yolk–shell microspheres with XRD and EPR analyses. (**B**) SEM images of Mn–Co-MOF (**a**,**b**) and Mn–Co–O@C (**c**,**d**); TEM image (**e**) and elemental mapping (**f**) of Mn–Co–O@C. (**C**) Electrochemical performance of Mn–Co–O@C: CV at 0.1 mV s^−1^, GCD at 0.5 A g^−1^, rate capability, and cycling stability at 0.5 and 2 A g^−1^. (**D**) CV curves at various scan rates, log(i)–log(v) analysis, and capacitive vs. diffusion-controlled contribution ratios of Mn–Co–O@C [55].

This study, Chengjie Yin et al., demonstrates how the inherent instability of Mn-based cathodes in aqueous Zn-ion batteries is addressed by carbon-coated manganese oxides derived from MOFs [55]. Figure 14A shows how carbon shells originating from MOF ligands effectively prevent Mn dissolution, improve conduction networks, and protect the Mn-oxide framework from structural breakdown. The architectural development is further clarified in Figure 14B(a–f), which illustrates how hierarchical MnO_x_@C flakes or nanosheets form multiple ion/electron transport channels, with the carbon layer serving as both a conductor and a buffer. By demonstrating how the carbon matrix stabilizes Mn oxidation states during cycling, reduces Jahn–Teller distortion, and promotes reversible Zn^2+^ intercalation, Figure 14C enhances structural understanding. High capacity, reliable performance over extended cycles, and preserved structure exemplify the exceptional electrochemical characteristics shown in Figure 14D, clearly indicating that combining surface carbonization with MOF-derived morphology is an effective strategy for robust Mn-based AZIB electrodes.

**Figure 15:**
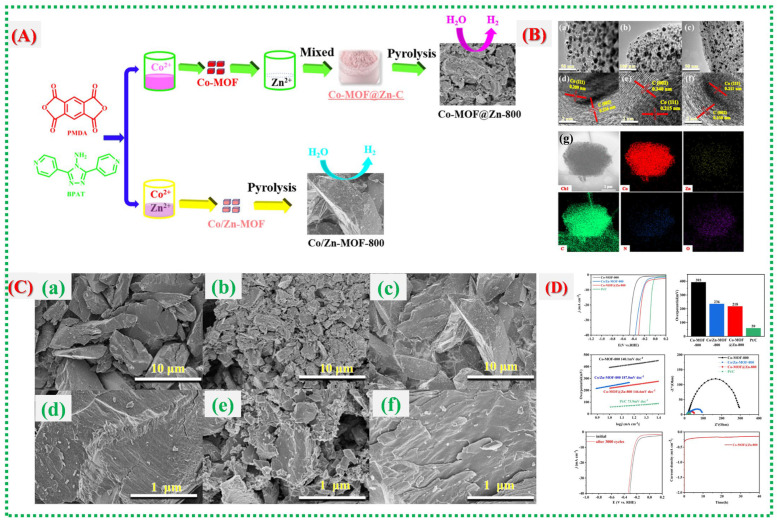
(**A**) shows the procedures for synthesizing Co-MOF@Zn-800 and Co/Zn-MOF-800. (**B**) High-magnification transmission electron microscopy (TEM) images of (**a**) Co-MOF-800, (**b**) Co-MOF@Zn-800, and (**c**) Co/Zn-MOF-800; high-resolution (HR) TEM images of (**d**–**f**); and energy dispersive spectroscopy (EDS) mapping of (**g**). (**C**) Also presented are SEM images of (**a**) Co-MOF-800, (**b**) Co-MOF@Zn-800, and (**c**) Co/Zn-MOF-800 at low magnification, with (**d**–**f**) at high magnification. (**D**) The figure includes data on overpotential at 10 mA cm^−2^, Tafel plots, EIS curves for Pt/C, Co-MOF@Zn-800, Co/Zn-MOF-800, and Co-MOF-800; LSV curves pre- and post-3000 CV cycles for Co-Mof@zn-800; and time-dependent current density retention curves [56].

In this study, Ling Qin et al. [56] examine a different MOF-derived composite system, emphasizing how multi-component integration and microstructural control can synergize to enhance electrochemical performance. Figure 15A outlines the formation mechanism, crystallinity confirmation, and uniform element distribution of the composite material, establishing a stable framework conducive to ion transport. Vigorous CV redox activity and GCD features in Figure 15B show improved reversibility and reduced polarization, which are indicative of the optimized internal pathways produced by the MOF-templated structure. Figure 15C provides detailed insights into surface chemistry and electronic states, suggesting that altered oxidation environments, lattice adjustments, or defect-engineered sites contribute to accelerated electron transport and improved reaction kinetics. The story is completed in Figure 15D, which shows device-level superiority, including high specific capacity, excellent rate capability, stable cycling behavior, and reduced impedance, proving that the MOF-derived architecture of the composite continuously promotes fast electrochemical kinetics and structural endurance. Recent reports on Co-MOFs and their derivatives are summarized in Table 3.

These three papers demonstrate that structural tunability, surface engineering, and interface optimization are consistently beneficial for MOF-derived Zn-ion battery cathodes, whether based on Co_3_O_4_–P systems, MnO_x_@C hybrids, or multi-component composites. Every figure reaches the same conclusion: modifying the surface environment and precursor chemistry enhances redox activity, improves Zn^2+^ transport, raises conductivity, and ensures long-term electrochemical stability. These insights provide a solid foundation for designing next-generation high-performance aqueous Zn-ion batteries.

The rational design of Mn-, Cu-, and Co-MOF-based electrodes increasingly depends on identifying reliable activity descriptors that link microstructure and electrical properties to Zn^2+^ storage performance. Common descriptors, such as orbital occupancy, metal–oxygen covalency, and d-band center, have been used to explain trends in oxygen electrocatalysis for transition-metal catalysts [68,69]. Similar principles can be applied to MOF-derived Zn-ion cathodes by examining factors such as the average oxidation state of Mn/Cu/Co centers, the stability of their coordination during cycling, the binding energies of Zn^2+^ and co-inserted species, and conjugation degrees in MOF-derived carbon frameworks. Recent machine learning studies on low-dimensional electrocatalysts suggest data-driven methods can identify subtle features and efficiently evaluate potential materials [70]. Integrating DFT calculations, operando characterization, and machine learning for MOF-based ZIB electrodes offers a promising approach to discover key descriptors such as Zn^2+^ diffusion barriers, free energies of reaction intermediates, and interfacial charge-transfer resistances, advancing the predictive design of Mn-, Cu-, and Co-MOF architectures.

### 3.4. V-MOF–Derived Materials for Zn-Ion Batteries

Vanadium-based metal–organic frameworks (V-MOFs) are highly promising precursors for advanced cathodes in aqueous ZIBs due to their inherent porosity, structural flexibility, and uniform metal distribution. When subjected to controlled thermal treatment, V-MOFs can be converted into porous or hierarchical V_2_O_5_ structures that preserve the original framework shape while adding numerous pathways for ion diffusion. MOF-derived V_2_O_5_ often exhibits mixed vanadium valence states (V^4+^/V^5+^), which boost electronic conductivity and lower polarization during Zn^2+^ insertion and extraction. Furthermore, the increased specific surface area and the micro- and mesoporous structures of V-MOFs greatly enhance electrolyte access and reaction kinetics. Consequently, V-MOF-derived cathodes exhibit superior specific capacity, rate performance, and cycle stability compared to bulk or commercial V_2_O_5_, making them strong candidates for next-generation aqueous zinc-ion batteries.

**Figure 16:**
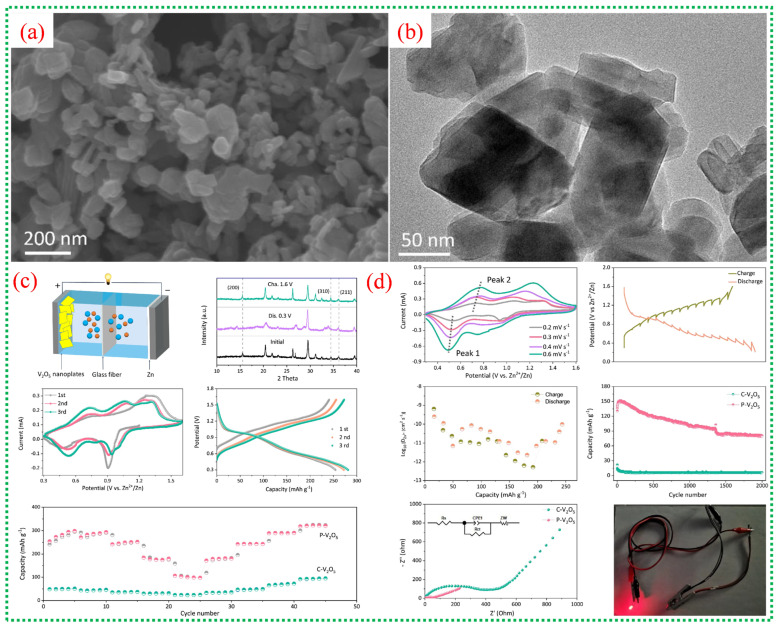
(**a**,**b**) reveal that V-MOF-derived V_2_O_5_ consists of ultrathin, porous nanoplates, as observed by SEM and TEM, providing numerous active sites and short diffusion paths for Zn^2+^ ions. (**c**) presents a schematic of the assembled aqueous Zn//V_2_O_5_ battery configuration. XRD analysis of V-MOFs, combined with cyclic voltammetry curves, shows two distinct redox couples corresponding to stepwise Zn^2+^ insertion and extraction alongside V^5+^/V^4+^/V^3+^ redox transitions. This further confirms a highly reversible Zn^2+^ intercalation mechanism, as reflected in stable electrochemical behavior and the beneficial effect of mixed vanadium valence states on charge storage kinetics. (**d**) illustrates that porous V_2_O_5_ delivers a substantially higher discharge capacity and lower polarization than commercial V_2_O_5_, while also demonstrating excellent cycling stability and Coulombic efficiency [71].

Figure 16a shows that the V-MOF-derived V_2_O_5_ consists of stacked ultrathin nanoplates, forming a porous, block-like structure that sharply contrasts the dense morphology of commercial V_2_O_5_. The TEM image in Figure 16b further confirms that these blocks are composed of thin nanosheets with lateral dimensions of several tens of nanometers. The shape of these ultrathin platelets shortens the diffusion pathways for Zn^2+^ and increases the contact area between the electrode and electrolyte, facilitating fast intercalation kinetics. Figure 16c presents a schematic of the aqueous Zn-ion battery configuration, with porous V_2_O_5_ as the cathode material. The CV curves in Figure 16c display two distinct sets of redox peaks, indicating multistep Zn^2+^ insertion/extraction processes and V^5+^/V^4+^/V^3+^ redox transitions. The electrochemical impedance spectra in Figure 16c show a lower charge-transfer resistance for the porous V_2_O_5_ electrode, indicating better electronic conductivity and faster interfacial kinetics than for commercial V_2_O_5_. Figure 16c,d shows galvanostatic charge–discharge profiles with clear voltage plateaus, indicating stable Zn^2+^ intercalation behavior. The rate performance in Figure 16d demonstrates that the porous V_2_O_5_ retains considerable capacity even at high current densities [71], highlighting the structural stability and rapid ion transport afforded by the MOF-derived porous architecture.

**Figure 17:**
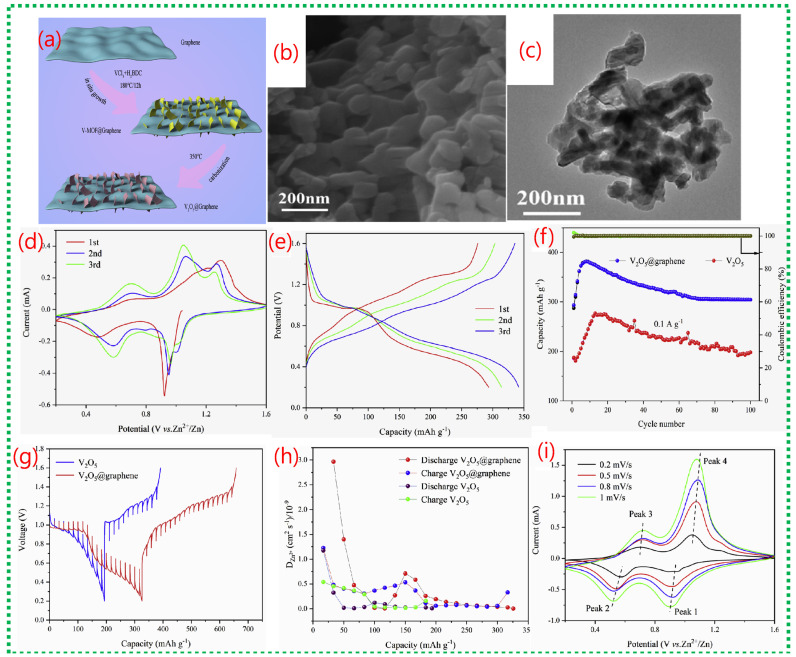
(**a**) Scheme illustrates the stepwise formation of a two-dimensional hierarchical V_2_O_5_@graphene composite. (**b**,**c**) show SEM and TEM images confirming that ultrathin V_2_O_5_ nanosheets are uniformly anchored on the graphene substrate. (**d**) presents CV curves with enhanced peak intensity and area, indicating improved Zn^2+^ storage kinetics due to graphene incorporation. (**e**,**f**) reveals higher reversible capacity and superior long-term cycling stability compared to bare V_2_O_5_. (**i**) shows CV curves at different scan rates, while (**g**) demonstrates b-value analysis, indicating a mixed diffusion-controlled and pseudocapacitive charge-storage mechanism. (**h**) confirms reduced charge-transfer resistance and stable electrode kinetics during prolonged cycling [71].

The scheme demonstrates how the 2D hierarchical V_2_O_5_@graphene composite forms, illustrating the in situ growth of V-MOF nanosheets on graphene surfaces, followed by heat treatment (Figure 17a). This approach ensures tight interfacial contact between ultrathin V_2_O_5_ nanosheets and conductive graphene, facilitating efficient electron transport and structural stability during cycling. Figure 17b,c confirm that the synthesized V_2_O_5_@graphene maintains a uniform two-dimensional nanosheet structure, evenly attached to the graphene sheets. This design minimizes nanosheet aggregation, exposes numerous active sites, and promotes quick Zn^2+^ diffusion, maximizing active material utilization. Figure 17d shows clear CV redox peaks linked to multivalent vanadium reactions, while Figure 17e indicates substantial reversible discharge capacities with minimal polarization. Long-term cycling in Figure 17f demonstrates excellent capacity retention and nearly 100% Coulombic efficiency, confirming the synergistic stabilizing effect of graphene combined with MOF-derived V_2_O_5_. Figure 17g–i reveal that charge storage involves both diffusion-controlled and pseudocapacitive processes [71], with a significant rise in capacitive contribution at higher scan rates.

**Figure 18:**
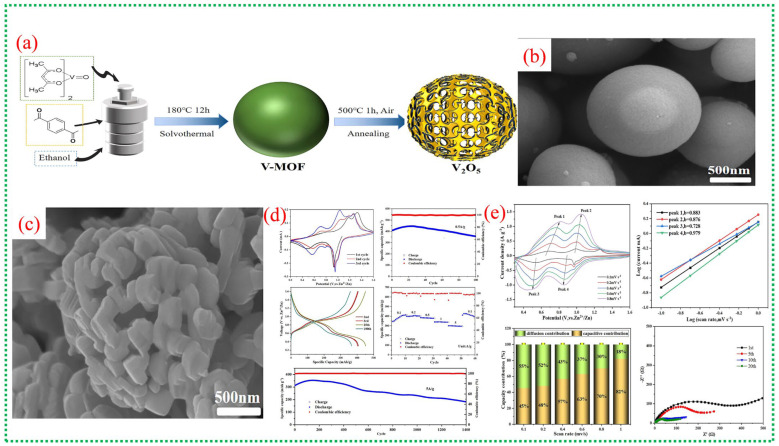
(**a**) schematically depicts the synthesis route, showing the conversion of spherical V-MOF precursors into skeletonized V_2_O_5_ microspheres via controlled oxidation. (**b**,**c**) show that the V-MOF precursor evolves into a porous, skeletonized V_2_O_5_ architecture composed of interconnected nanorods. (**d**) comprehensively demonstrates the electrochemical performance, including CV profiles, cycling stability, rate capability, and charge–discharge behavior. The skeletonized V_2_O_5_ delivers ultrahigh capacity and outstanding durability even at high current densities. (**e**) The figure analyses Zn^2+^ storage kinetics using scan-rate-dependent CV and impedance spectroscopy. The results reveal a dominant capacitive contribution at high scan rates and low charge-transfer resistance, confirming fast reaction kinetics enabled by the porous skeleton framework [72].

Figure 18a illustrates the synthesis process from spherical V-MOF precursors to skeletonized V_2_O_5_ microspheres via controlled oxidation. This method maintains the overall spherical shape while creating an internal porous structure that facilitates electrolyte penetration and provides strain relief during cycling. In Figure 18b, the pristine V-MOF appears as smooth, uniform microspheres, whereas Figure 18c shows that after calcination, these spheres evolve into interconnected porous frameworks made of short V_2_O_5_ nanorods. This skeletal structure greatly enhances the surface area and reduces transport distances for Zn^2+^ ions. Figure 18d provides an in-depth view of the electrochemical performance of skeletonized V_2_O_5_, with CV curves showing multiple redox peaks, notable reversible capacities at moderate currents, excellent rate capability, and long-term cycling stability [72]. The data underscore the kinetic benefits of the three-dimensional porous architecture and the mixed valence states of V^4+^ and V^5+^. Figure 18e investigates the charge-storage mechanism, highlighting a significant pseudocapacitive contribution alongside diffusion-controlled Zn^2+^ storage. Electrochemical impedance results further demonstrate low internal resistance and stable electrode–electrolyte interfaces, confirming the effectiveness of MOF-derived skeletonized structures for high-performance zinc-ion batteries. A comprehensive overview of recent literature on V-MOFs and their derivatives is presented in Table 4.

## 4. Conclusions

This study reviews recent developments in Mn-, Cu-, and Co-based metal–organic frameworks (MOFs) and their derivatives for aqueous zinc-ion batteries. It emphasizes that these three MOF systems are more practically significant and possess greater electrochemical potential than many other documented MOFs. Their varied coordination environments, multiple redox-active sites, and ease of conversion into functional derivatives—including oxides, sulfides, phosphates, and carbon composites—make them particularly suitable for electrode applications. A detailed comparison of current studies, organized in separate tables for Mn-, Cu-, and Co-MOFS, highlights the notable progress achieved through structural engineering, heteroatom integration, nanostructuring, and composite synthesis.

While there is potential for enhanced performance, several obstacles remain. These include structural instability during repeated Zn-ion insertion and extraction, capacity loss due to dissolution or conversion reactions, limited electronic conductivity in unmodified MOFs, and difficulties in understanding their charge-storage mechanisms. Overcoming these challenges requires detailed mechanistic studies, advanced in situ characterization techniques, and the strategic design of hierarchical nanoarchitectures. Mn-, Cu-, and Co-based MOFs are promising candidates for advancing aqueous zinc-ion battery technology. Further research into their derivatives, improved synthesis methods, and the development of multifunctional hybrid frameworks will be crucial for creating high-energy, durable, and cost-effective ZIBS.

## Figures and Tables

**Table 1:** Mn-MOFs and Mn-MOF-derived Composites for Aqueous Zn-Ion Batteries.

Mn-MOF/Composite	Rate (C or mA g^−1^)	Reversible Capacity (mAh g^−1^)	Cycle Number/Remark	Reference
Mn(BTC) (Mn-1,3,5-benzenetricarboxylate)	50 mA g^−1^	112 mAh g^−1^	900 cycles	[28]
MOF-73 (Mn-based)	50 mA g^−1^	815 mAh g^−1^ (peak after activation)	1000 cycles at 0.3 A g^−1^ → ~137 mAh g^−1^	[2]
Coordinately unsaturated Mn-MOF	100 mA g^−1^	138 mAh g^−1^	1000 cycles at 3000 mA g^−1^ (93.5% retention)	[31]
Zn/Mn-MOF-74 (double-redox design)	100 mA g^−1^	252.6 mAh g^−1^	1000 cycles	[32]
Mn-MOF/CNT composite	50 mA g^−1^	~260 mAh g^−1^	900 cycles @ 1000 mA g^−1^	[33]
MOF-derived Mn_3_O_4_@C hierarchical nanospheres	0.2 A g^–1^	331.5 mAh g–1	2000 @ 3 Ag^−1^	[34]
Se-doped MnS/Ti_3_C_2_Tx (MOF-derived)	1.0 A·g^−1^	50.6 mAh·g^−1^	500 cycles	[35]
MnO_2_–MOF-74 composite (in situ)	50–200 mA g^−1^	355.4 mAh g^−1^	400 @ 0.5 A·g^−1^	[36]
Mn-BTC-derived Mn_3_O_4_/C	200 mA/g	430 mAh/g	40 cycles @ 100 mA g^−1^	[37]
Mn-MOF derived composites with Ti_3_C_2_ (MXene) or rGO	50 mA g^−1^	264 mAh/g	100 cycles @1 Ag^−1^	[38]

**Table 2:** Cu-MOFs and Cu-MOF-derived/Cu-based materials relevant to aqueous Zn-ion batteries.

Material (Cu-MOF or Cu-Derived)	Rate (C or mA g^−1^)	Reversible Capacity (mAh g^−1^)	Cycle	Reference
CuS (activated CuS cathode)	0.2 A g^−1^	≈800 mAh g^−1^	1000 cycles	[42]
Hierarchical CuS hollow spheres (anode)	0.1–1 A g^−1^	126 mAh g^−1^	1500 cycles	[43]
Cu_0.26_V_2_O_5_@C (Cu-MOF-derived vanadium oxide)	0.1–1 A g^−1^	328.8 mAh g^−1^	500 cycles	[40]
CuO-containing composites (e.g., ZnMn_2_O_4_/CuO)	300 mA g^−1^	131.7 mAh g^−1^ at 500 mA g^−1^	100 cycles	[44]
HKUST-1 coating (Cu-MOF) for Zn anode protection	20 mA/cm^2^	114 mAh/g	500 cycles	[45]
Cu_3_(HHTP)_2_ (2D conductive Cu-MOF)—potential MOF cathode	4000 mA g^−1^	228 mAh g^−1^	500 cycles	[46]
Cu_2_O-CDs composite electrode (Cu_2_O-based cathode)	0.1 A g^−1^	425 mA h g^−1^	100 cycles	[47]
CuS@C composites (carbon coated CuS)	0.1 A g^−1^	225.3 mA h g^–1^	3400 cycles	[48]
Cu_7_S_4_ hollow structures (porous)	1 Acm^− 2^	1110.65 Fcm^− 2^	2000 cycles	[49]
Cu-doped V_2_O_5_ (MOF-derived) variants	100 mA·g^−1^	430 mAh·g^−1^	1000 cycles	[50]
Cu_2_V_2_O_7_@C nanofilm (MOF-derived)	5 A g^−1^	178 mAh g^−1^	2000 cycles	[51]
Cu-based PBA/CuS hybrid (MOF-derived templates used in some studies)	2 A g^−1^	113 mAh g^−1^	400	[52]
CuS-te substituted (Te-doped CuS)	1 A g^−1^	446 mAh g^–1^	1500 cycles	[53]

**Table 3:** Co-MOFs and Co-MOF-derived Composites for Aqueous Zn-ion Batteries.

Material	Rate	Capacity (mAh/g)	Cycle Life	Reference
Mo-Co_3_O_4_-CNTc	0.5 A g^−1^	195.7 mAh g^−1^	10,000 cycles (85%)	[57]
Co_3_O_4_/α-MnO_2_	0.4 C	243.5 mAh g^−1^	2000 cycles	[58]
O-vacancy Co_3_O_4_	1 mA cm^−2^	711 mAh g^−1^	600 cycles	[59]
Zn-doped Co_3_O_4_	1 A g^−1^	196 mA h/g	1000 cycles (93.2%)	[60]
ZIF-67 Co_3_O_4_ nanosheets	0.1–1 A g^−1^	203 mAh g^−1^	1000 cycles (82%)	[61]
Co_3_O_4_/rGO nanosheets	0.5 A g^−1^	175 mAh g^−1^	2000 cycles	[62]
CoNi_2_O_4_ nanosheet arrays	0.5 A g^−1^	221 mAh g^−1^	5000 cycles (92%)	[63]
CoS_2_ hollow nanocages	1 A g^−1^	268 mAh g^−1^	1500 cycles	[64]
Co_2_(OH)_3_Cl/CoOOH	0.2 A g^−1^	305 mAh g^−1^	500 cycles	[65]
Co_3_O_4_@MnO_2_ core–shell	0.2 A g^−1^	287 mAh g^−1^	1000 cycles	[66]
CoN@C composite	0.1 A g^−1^	158 mAh g^−1^	1000 cycles	[67]

**Table 4:** Vanadium-based materials for Li-ion batteries.

Material (Form/Example)	Rate (C or mA g^−1^)	Specific Capacity (mAh g^−1^)	Cycle/Remark	Reference
V_2_O_5_ (yolk–shell, nanoplate)	1000 mA g^−1^	271 (initial)/201 after 100 cycles	100 cycles: 201 mAh g^−1^ (at 1000 mA g^−1^)	[73]
LiV_3_O_8_ (nanosheets)	100 mA g^−1^	260	No capacity fading over 100 cycles (100 mA g^−1^)	[74]
Li_3_V_2_(PO_4_)_3_ (r-LVP, LISICON-structured)	up to 10C (high-rate tests)	≈142–163 (typical intercalation range)	5000 cycles at 10 C: 84% retention	[75]
VO_2_(B) (Al-doped nanobelts)	32.4 mA g^−1^	282 (initial) → 202 after 50 cycles	50 cycles: 202 mAh g^−1^ (32.4 mA g^−1^)	[76]
V_2_O_5_–graphene (graphene-modified V_2_O_5_ hybrids)	0.05 C	≈438	200 cycles@1 C	[77]
V_2_O_3_ (polycrystalline nanorods)	0.4 C	≈195 (2nd cycle) → ~120 after long cycling	Retains ~120 mAh g^−1^ after 750 cycles (0.4 C)	[78]
Li_5_V_3_O_8_ (high-rate anode)	40 C	152 (40 C)	80% retention after 11,000 cycles at 20 C	[79]
V_6_O_13_	1 C	≈306 (at 1 C)	50 cycles @ 0.1 C	[80]

## Data Availability

No new data were created or analyzed in this study. Data sharing is not applicable to this article.

## Abbreviations

C	Specific capacitance (F g^−1^ or mF cm^−2^)
E	Energy density (Wh kg^−1^)
P	Power density (W kg^−1^)
V	Voltage (V)
I	Current (A or mA)
Q	Charge or capacity (C or mAh g^−1^)
t	Time (s or h)
R	Resistance (Ω)
σ	Electrical conductivity (S cm^−1^)
η	Efficiency (%)
d	Thickness or diameter (nm or µm)
